# Clinical trial landscape of cellular therapies in bladder cancer

**DOI:** 10.1097/JS9.0000000000004060

**Published:** 2025-12-16

**Authors:** Yang Zhang, Shuangwu Lv, Zhijun Li, Xiaohui Wang, Yanping Zhu

**Affiliations:** Department of Urology Surgery, The First Affiliated Hospital, and College of Clinical Medicine of Henan University of Science and Technology, Luoyang, China

Bladder cancer (BC) poses a significant global health burden, ranking among the top ten malignancies worldwide with high recurrence and progression rates^[1]^. Current interventions for nonmuscle-invasive and muscle-invasive disease impose substantial morbidity and costs, while therapeutic outcomes remain suboptimal^[2]^. Cellular therapies represent promising strategies to overcome the immunosuppressive tumor microenvironment and antigen heterogeneity in BC^[3]^. Their potential motivates this systematic analysis of the global clinical trial landscape. Characterizing trial phases, targets, and geographic distributions identifies research priorities to accelerate clinically impactful cellular therapies for BC^[4]^. This research letter provides a detailed overview of the current status and future directions of cell therapy clinical trials in BC and practical insights for stakeholders.

We comprehensively searched the Trialtrove database (accessed June 2025) using the query terms “(Disease is Oncology: Bladder) AND (Drug Type is Biological > Cellular).” Analyzed metrics contained temporal trends of clinical trials, trial phases/status, drug target, classification of cell therapy, sponsor types, and geographic distribution. A total of 277 trials were searched, and 170 eligible trials were included. Detailed retrieval categories are provided in S1. Two individual investigators independently rechecked the source data, including data extraction and data integration, and confirmed the accuracy of the analyses, ensuring that relatively tight quality control was achieved. After processing through the quality control procedure, residual limiting factors do not affect the core analytical trend of the study.^[5]^ And we ensure that our study is compliant with the TITAN Guidelines 2025^[6]^.

A total of 170 eligible trials were identified eventually (Supplemental Digital Content Table S1, available at: http://links.lww.com/JS9/F884). The time analysis indicates that from 1995 to 2025, the number of trials increased, with phase I clinical studies being dominant, followed by phase II trials (Fig. 1A). Most of the trials have been completed, and 19 trials are currently open (Fig. 1B), in known Terminated trials, Terminated, Planned but never initiated accounted for the majority (26%), followed by Terminated, Business decision – Other (20%) (Supplemental Digital Content Table S1, available at: http://links.lww.com/JS9/F884). In terms of the sponsor type, academic institutions sponsored 43.1% of the trials, while industry-sponsored trials accounted for 36.9% (Fig. 1C, Supplemental Digital Content Table S1, available at: http://links.lww.com/JS9/F884). Geographically, 32.3% of the trials were conducted in the United States, followed by Asia, with limited trials from South America and Australia (Fig. 1D), which is consistent to some extent with the differences in national disease burdens and medical systems. For the primary endpoints, safety and tolerability and adverse events are the two most important indicators (Fig. 1E). Cell therapy can be divided into three categories, of which cell type accounted for 63.3% of the described test types, of which bacterial cells accounted for 65.4% of the total. This was followed by cell origin, which accounted for 22.3% of all illustrated trials, (defined as using the patient’s own cells as the source for treatment^[7]^.) was more than half (52.6%). Cell technology type accounts for only 14.4% (Fig. 1F). Although most research targets are not applicable (Fig. 1G) or not specified, among the specified targets, programmed cell death 1 and interleukin 15 receptor subunit alpha are commonly used.

**Figure 1.**
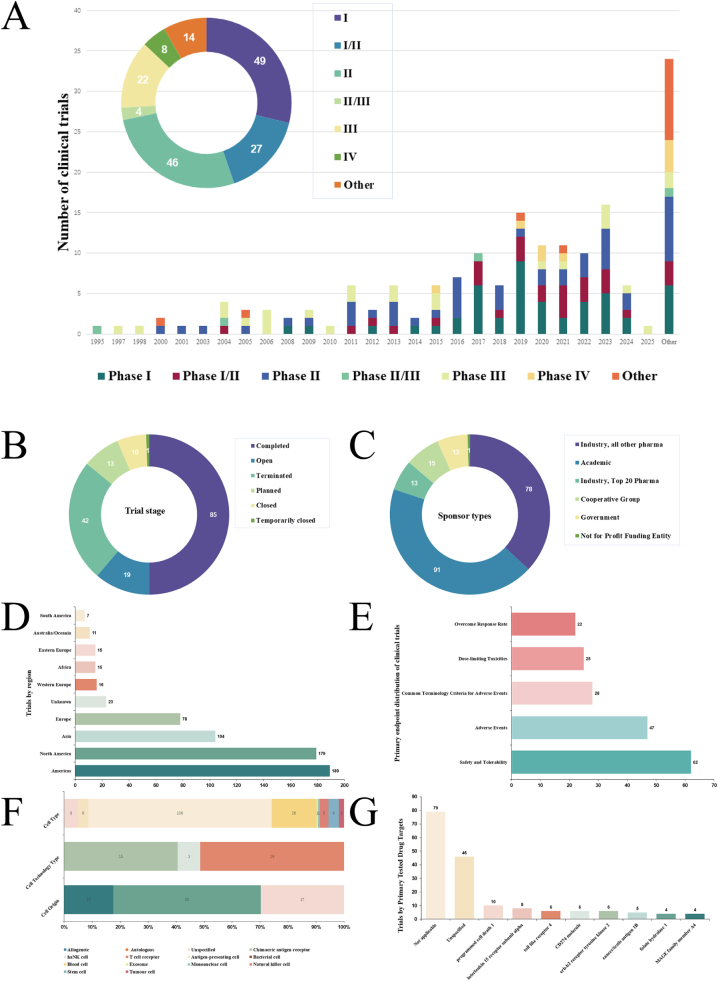
The panorama of clinical trials for cell therapy for bladder cancer. (A) Time trend and phase distribution of clinical trials; (B) Trial stage distribution of clinical trials; (C) Sponsor type distribution of clinical trials; (D) Country distribution of clinical trials (Top 10); € Primary endpoint distribution of clinical trials (Top 5); (F) Classification of cell therapy; (G) Trials by primary tested drug targets (Top 10).

Emphatically, the proliferating clinical trial landscape demonstrates transformative potential of cellular immunotherapies in reshaping BC management. Notably, engineered “armored” CAR-T constructs and CAR-NK/NKT platforms leverage precision immune targeting against oncogenic axes, with China’s regulatory innovation accelerating trial initiation^[4]^. Landmark FDA approvals of TIL (lifileucel)^[8]^ and TCR (afamitresgene autoleucel) therapies for solid tumors validate the translatability of cellular approaches, providing critical proof-of-concept for BC applications^[9]^. Simultaneously, NK-cell strategies and oncolytic viruses capitalize on innate immune remodeling, demonstrating preclinical efficacy against urothelial malignancies. Nevertheless, CAR-T/CAR-NK technologies face pivotal translational hurdles: defining urothelial-specific antigens remains imperative to mitigate on-target/off-tumor toxicity, while optimizing combinatorial regimens with checkpoint blockade or targeted therapy is essential to enhance antitumor potency^[9,10]^. At the same time, antigen discovery in bladder cancer should prioritize patient-specific neoantigens and validated shared antigens to maximize specificity. Antigen discovery should give priority to MUC1 and HER2, with > 40% expression in bladder tumors. In early trials, Combination strategies such as CAR-T plus PD-1 blockade leading to response rates as high as 38% should move from biomarker-rich phase I trials to randomized phase II studies with prespecified durability and response rate endpoints, allowing for rigorous, data-driven advances in regulatory transparency and cost-effectiveness metrics^[11].^ Critically, although no cellular product has yet gained BC approval, 277 active trials – predominantly industry–academia partnerships – signal concentrated investment in overcoming these barriers. The EMA’s ATMP frameworks and FDA’s 2024 CAR-T guidance now mandate rigorous clinical validation, urging alignment of innovation with unmet clinical needs^[3]^. Similarly, robust preclinical validation, adaptive designs with early futility checks, and sustainable funding strategies are needed for future cell therapy development in bladder cancer to reduce premature termination. Despite prevailing challenges of early-phase trial dominance, current breakthroughs collectively underscore the strategic value of cellular platforms. TIL therapy’s proven capacity to overcome solid tumor barriers^[8]^, complemented by CAR-NK’s nonviral manufacturing advances, positions these modalities as vanguards for BC treatment paradigms. Harmonizing technological innovation with biomarker-driven patient selection will catalyze the transition from experimental pipelines to clinical impact^[9]^.

In summary, the global trial scope of BC cell therapy is expanding rapidly, but it is still in its early stages. This analysis highlights the dispersion of the development process of cell therapies for BC, characterized by regional differences, dependence of sponsors on academia, and the need for diverse mechanisms. Future work must prioritize global cooperation, adaptive trial design, and integration of preclinical breakthroughs of specific therapies and organ-specific damage to improve clinical outcomes.

## Supplementary Material

## Data Availability

Yes.

## Availability of data and materials

All data for this study have been provided.
